# Vascular endothelial injury exacerbates coronavirus disease 2019: The role of endothelial glycocalyx protection

**DOI:** 10.1111/micc.12654

**Published:** 2020-08-30

**Authors:** Hideshi Okada, Shozo Yoshida, Akira Hara, Shinji Ogura, Hiroyuki Tomita

**Affiliations:** ^1^ Department of Emergency and Disaster Medicine Gifu University Graduate School of Medicine Gifu Japan; ^2^ Department of Tumor Pathology Gifu University Graduate School of Medicine Gifu Japan

**Keywords:** acute respiratory distress syndrome, COVID‐19, glycocalyx, systemic disease, thrombosis

## Abstract

The potential for a rapid increase in severity is among the most frightening aspects of severe acute respiratory syndrome coronavirus 2 infection. Evidence increasingly suggests that the symptoms of coronavirus disease‐2019 (COVID‐19)‐related acute respiratory distress syndrome (ARDS) differ from those of classic ARDS. Recently, the severity of COVID‐19 has been attributed to a systemic, thrombotic, and inflammatory disease that damages not only the lungs but also multiple organs, including the heart, brain, toes, and liver. This systemic form of COVID‐19 may be due to inflammation and vascular endothelial cell injury. The vascular endothelial glycocalyx comprises glycoproteins and plays an important role in systemic capillary homeostasis maintenance. The glycocalyx covers the entire vascular endothelium, and its thickness varies among organs. The endothelial glycocalyx is very thin in the pulmonary capillaries, where it is affected by gaseous exchange with the alveoli and the low intravascular pressure in the pulmonary circulation. Despite the clearly important roles of the glycocalyx in vascular endothelial injury, thrombosis, vasculitis, and inflammation, the link between this structure and vascular endothelial cell dysfunction in COVID‐19 remains unclear. In this prospective review, we summarize the importance of the glycocalyx and its potential as a therapeutic target in cases of systemic COVID‐19.

**Abbreviations**
ACE2: angiotensin‐converting enzyme 2
AE: venous thromboembolism
ARDS: acute respiratory distress syndrome
COVID‐19: coronavirus disease‐2019
ICU: intensive care unit
LPS: lipopolysaccharide
rhTM: recombinant human thrombomodulin
SARS‐CoV‐2: severe acute respiratory syndrome coronavirus 2
TF: tissue factor
TFPI: tissue factor pathway inhibitor
VTE: venous thromboembolism

## 1 INTRODUCTION

Reports continue to describe the biological, epidemiological, and clinical characteristics of infection with the SARS‐CoV‐2 and the related disease, coronavirus infection 2019 COVID‐19. To date, however, information about the clinical characteristics of patients with COVID‐19 who require ICU care remains inadequate or uncertain. A few recent reports have described some characteristics of severe cases of this disease. One study reported that patients in Wuhan, China, who were older than 65 years and presented with comorbidities and ARDS had a higher mortality rate and survival durations of 1‐2 weeks after admission to the ICU.^1^ Another study determined that most patients with confirmed COVID‐19 who were admitted to an ICU in Lombardy, Italy, were elderly, required mechanical ventilation, had high positive end‐expiratory pressure levels, and ultimately had an ICU mortality rate of 26%.^2^

COVID‐19‐related ARDS is the main cause of SARS‐CoV‐2‐triggered mortality. Typically, ARDS is characterized by an influx of fluid into the lungs, such that breathing becomes impossible and the patient's oxygenation levels plunge. ARDS can only be cured with time, and artificial respiration is required until the inflammatory fluid subsides. However, the reports of COVID‐19 ARDS in ICU departments differ from those of typical ARDS.^3^ Moreover, a recent autopsy report of three patients with COVID‐19 demonstrated that SARS‐CoV‐2 infection induced endotheliitis in several organs, including the ARDS‐affected lungs, both as the direct result of viral involvement (as noted by the histological presence of viral bodies) and the inflammatory response of the host.^4^ In other words, aggravated COVID‐19 involves vascular endothelial cells in different organs throughout the human body.

Endothelial dysfunction unbalances the vascular equilibrium to favor vasoconstriction, with subsequent organ ischemia, inflammation with associated tissue edema, a pro‐coagulant state, and is a major determinant of microvascular dysfunction. The capillary, which is also referred to as a microvascular endothelial cell, has a diameter of 5‐20 µm. The exchange of various molecules, including oxygen, between the blood and organs only occurs via the capillaries. Accordingly, the capillaries play a central role in systemic microcirculation. The inner surface of all vascular endothelium is coated by the vascular endothelial glycocalyx, which comprises cell‐bound proteoglycans, glycosaminoglycan side chains, and sialoproteins. This endothelial glycocalyx plays an important role in microvascular and endothelial physiology.^5^, ^6^, ^7^, ^8^ We propose that thrombosis may be associated directly with both the onset and exacerbation of COVID‐19 (eg, ARDS, heart failure, cerebral infarction) via the endothelial glycocalyx, which would serve as the missing link in the complex pathogenesis of this disease. Here, we review the possible roles of the endothelial glycocalyx in COVID‐19 and propose targeted therapeutic strategies.

## 2 HOW DOES COVID‐19 ARDS DIFFER FROM CLASSIC ARDS?

Patients with COVID‐19 are at risk of developing ARDS. The best course of treatment for COVID‐19 ARDS remains controversial, particularly as accumulating evidence suggests that COVID‐19 ARDS differs from classic ARDS. Like classic ARDS, progressive COVID‐19 ARDS is characterized by protein‐rich edema and fibrin debris in the lungs. Moreover, COVID‐19 is unique with respect to the presence of a lymphocytic and mononuclear cell infiltration of the lungs and a much higher fatality rate of 70% or even 80%, compared to 40% with classic ARDS.

Many patients with COVID‐19 develop severe hypoxemia but exhibit normal respiratory compliance (ie, the ability to dilate the lungs when breathing in air). Although such patients require oxygen, they do not need high‐pressure ventilation. In contrast, patients with typical ARDS, such as that caused by influenza, usually find it very difficult to breathe and require intubation because of severe hypoxia. In such patients, the lung compliance decreases as the severity of the gas exchange abnormality worsens. Gattinoni et al^3^ has described two distinct phenotypes of COVID‐19 ARDS: Type L and Type H. Patients typically present with Type L disease, which is characterized by a normal lung compliance and gas volume despite the presence of hypoxemia. The condition of these patients tends to improve. However, approximately 20%‐30% of patients present with or evolve to Type H disease, which is characterized by poor lung compliance and increases in edema and lung weight. The transition from Type L to Type H may be attributable both to the evolution of COVID‐19 pneumonia and the injuries caused by high‐stress ventilation.

One report published in early 2020 proposed that COVID‐19‐related lung injury is similar to high‐altitude pulmonary edema,^9^ a life‐threatening form of non‐cardiogenic pulmonary edema that typically occurs in lowlanders who ascend rapidly to altitudes greater than 2500‐3000 m.^10^ Certainly, these entities share some clinical features, such as hypoxemia, radiographic opacities, and altered lung compliance. However, the underlying pathophysiological mechanisms are fundamentally different, and the entities cannot be viewed as equivalent.^11^

Although histopathological data are sparse, needle biopsies of the lungs in four patients with fatal COVID‐19 pneumonia mainly revealed the presence of hyaline membrane formation, fibrin exudates, epithelial damage, and diffuse type II pneumocyte hyperplasia, all of which are features of diffuse alveolar damage.^12^ Mild alveolar wall thickening was also evident in some cases, which suggested a more advanced stage of disease. However, these cases did not exhibit mature fibrosis, unlike previous cases of SARS and Middle East respiratory syndrome.^12^ Taken together, accumulating evidence indicates that COVID‐19 ARDS and classic ARDS differ both clinically and histologically.

## 3 SYSTEMIC INFLAMMATION AND MULTIPLE THROMBOSIS AS A MAJOR PATHOGENIC MECHANISM OF COVID‐19 ARDS AND OTHER DISEASES

SARS‐CoV‐2 is presumed to multiply in alveolar epithelial cells, which express the ACE2 receptor. In this setting, the virus causes lung damage while simultaneously infecting alveolar macrophages and inducing local inflammation. Subsequently, the main cause of COVID‐19 ARDS is attributed to an immune system collapse, or “cytokine storm,” which leads to severe ARDS and destroys the lung cells.^13^ ARDS has been recognized as a lethal complication of COVID‐19 since the beginning of the associated pandemic. Although COVID‐19 was initially believed to be a respiratory disease, increasing evidence suggests that organs such as the kidneys, heart, liver, and brain are also affected.^13^ SARS‐CoV‐2 initially targets the lungs; however, in severe cases, this virus can cause major systemic damage in the lungs, heart, brain, eyes, nose, liver, kidneys, intestines, and skin.^13^

Recently, clinicians and researchers have questioned whether endothelial disorders may cause early thrombosis, which would later complicate ARDS. An earlier study of 183 COVID‐19 patients in China found small blood clots throughout the systemic blood vasculature in 71% of those who died.^14^ Subsequent reports of COVID‐19‐associated thrombosis suggest that blood clots are a major contributor to disease severity and mortality. For instance, in 184 ICU patients, about one‐third had VTE 27%), AE (3%), pulmonary or leg thrombosis, and in many cases, stroke.^15^ Magro and colleagues identified blood clots in the lungs of two patients who died of COVID‐19, as well as blood clots on the inside of the skin (including the palms and feet) in three COVID‐19 survivors.^16^ The latter phenomenon, also known as “COVID toes,” is characterized by a frostbite‐like red or purple appearance, and it is more common in children and young people with less severe symptoms. In addition, a purple mesh‐like pattern may appear on the skin. Both phenomena are suspected to be related to blood clots.

Recent autopsy studies of COVID‐19 patients revealed that SARS‐CoV‐2 infects the vascular endothelial cells and causes endotheliitis in many organs throughout the body, including the lung, intestine, liver, kidney, and stomach.^4^ Moreover, the National Health Service in the United Kingdom reported the occurrence of a multisystem inflammatory disease similar to Kawasaki disease in pediatric COVID‐19 patients. Specifically, this phenomenon is characterized by inflammation of the walls of the blood vessels, including arteries, veins, and capillaries, throughout the body.^17^ Taken together, these results indicate that COVID‐19 is a systematic disease related to thrombosis and endothelial dysfunction, as well as inflammation. However, it remains unclear whether the virus directly triggers the pro‐coagulation cascade or whether other mechanisms are involved. The virus induces systemic inflammation and may thus cause lesions that affect the vasculature. However, viral infection may directly cause vasculitis, or inflammation of the vessel walls.

Microvascular disorders are difficult to diagnose via imaging modalities. Moreover, the condition may be misdiagnosed because the initial cardiovascular abnormalities occur in microvessels. Accordingly, serum biomarkers specific for COVID‐19 complications are needed. One study suggests that the serum D‐dimer concentration may be predictive of mortality in COVID‐19 patients; specifically, the D‐dimer concentration appears to be an early and useful determinant of the future progression to severe disease.^18^ However, the search for biomarkers remains at its early stages.

## 4 ENDOTHELIAL GLYCOCALYX: THE GATEKEEPER OF VASCULAR HOMEOSTASIS

**Figure 1:**
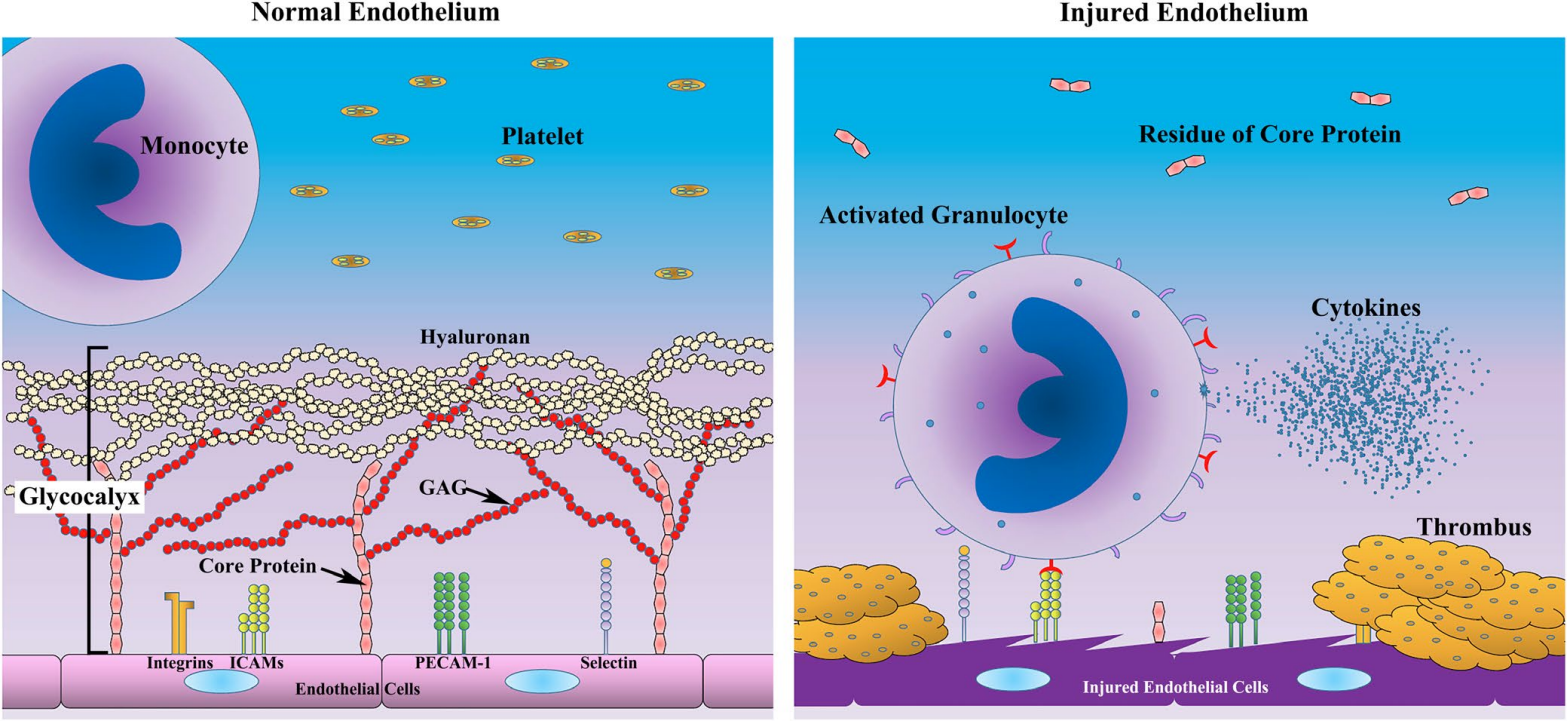
Schema of the endothelium. The surface and surface receptors of the normal endothelium are covered by the endothelial glycocalyx, which is composed of the core protein, GAG, and hyaluronan. Only the core protein binds to the endothelial cells, whereas GAG and hyaluronan do not directly interact with these cells (Left Panel). However, the endothelial glycocalyx is degraded under conditions of injury, such as the cytokine storm. Here, both the surfaces and surface receptors of endothelial cells are exposed to the vascular lumen. Granulocytes and platelets adhere to the endothelial cells, causing injury and thrombi, which block the blood flow (Right Panel). GAG: Glycosaminoglycan

The sugar‐protein glycocalyx plays a key role in microvascular and endothelial physiology. This structure contributes to the regulation of microvascular tone and endothelial permeability, maintenance of an oncotic gradient across the endothelial barrier, regulation of leukocyte adhesion/migration, and inhibition of intravascular thrombosis.^5^, ^6^, ^7^, ^8^, ^19^ The glycocalyx comprises of cell‐bound proteoglycans, which consist of a core protein (eg, syndecan family protein), as well as glycosaminoglycan side chains and sialoproteins.^20^, ^21^ The glycosaminoglycan side chains create a high density of negative charges, which drive albumin away from the vessel wall and toward the lumen via electrostatic repulsion.^22^ The intact glycocalyx prevents the inadvertent adhesion of platelets and leukocytes to the vascular wall.^7^, ^23^, ^24^, ^25^ Specifically, the glycocalyx thickness of approximately 0.5 µm exceeds the dimensions of cellular adhesion molecules expressed on endothelial cells and thus attenuates the interactions of these molecules with circulating blood cells (Figure 1).^24^, ^25^

**Figure 2:**
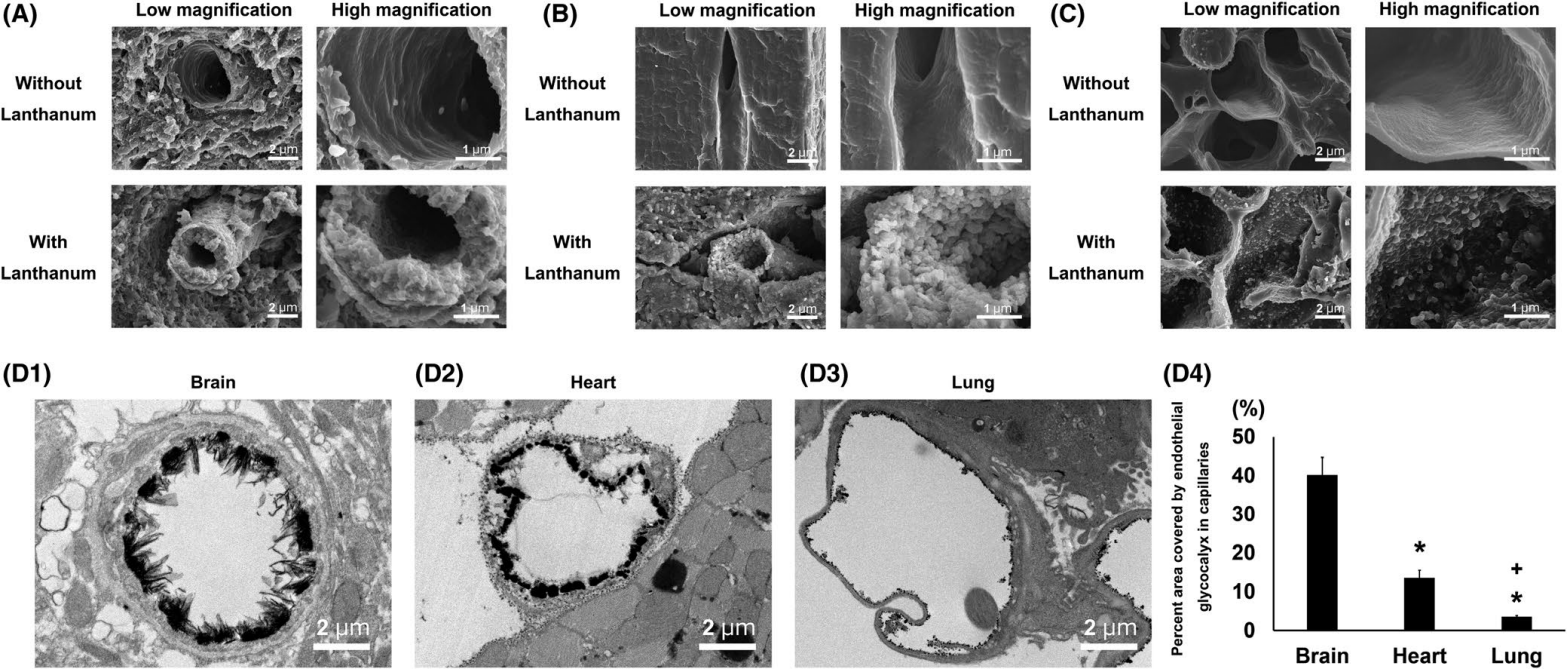
Scanning electron micrographs depicting the ultra‐structures of continuous capillaries in the (A) brain, (B) heart, and (C) lung. The upper and lower panels depict micrographs without and with lanthanum nitrate staining for the visualization of the endothelial glycocalyx, respectively. The panels on the right are the expanded views of each panel on the left. Continuous capillaries have a continuous basement membrane, and the endothelial glycocalyx is visible on the surfaces of the vascular endothelial cells. (D) Transmission electron microscopic analysis of continuous capillaries. Cerebral (D1), cardiac (D2), and pulmonary (D3) capillaries with lanthanum nitrate staining. The endothelial glycocalyx is seen to cover the surfaces of the vascular endothelial cells. (D4) Percent area covered by the endothelial glycocalyx in the capillaries of the brain, heart, and lung. The bars indicate the means ± standard errors. * and ^+^ represent *P* < .05 vs the brain and heart, respectively (Ref.^27^)

The endothelial glycocalyx coats the surface of all healthy endothelial structures. However, the morphology of the glycocalyx varies among the different types of capillaries. In continuous capillaries, the endothelial glycocalyx exhibits a moss‐ or broccoli‐like appearance and is distributed over the entire luminal surface of the endothelium. In fenestrated capillaries, the glycocalyx appears to nearly occlude the endothelial pores. In sinusoidal capillaries, the glycocalyx does not occlude the open fenestrations and is thinner than those observed in continuous or fenestrated capillaries.^26^ Moreover, a recent study revealed that the endothelial glycocalyx in the brain differs from that in the heart and lung, despite the fact that all three organs contain continuous capillaries (Figure 2).^27^ According to that study, the pulmonary capillaries were injured more easily than the cardiac and cerebral capillaries, and this difference was attributed to a relatively thinner endothelial glycocalyx in the lungs.

## 5 ENDOTHELIAL GLYCOCALYX INJURY IN SEVERAL SITUATIONS

Disruption of the glycocalyx exposes the endothelial cells to oxidative damage. Vascular hyperpermeability is observed in sepsis, a condition defined by infection and organ failure,^28^ as well as in chronic conditions such as diabetes and hypertension.^6^, ^29^ Systemic inflammation, such as sepsis, causes endothelial dysfunction, which leads to increased paracellular permeability and albumin/fluid outflow into the interstitial space.^8^ Potentially, glycocalyx disruption might cause these phenomena. Previous reports also suggested that the degradation of the endothelial glycocalyx contributes to the pathogenesis of ARDS, a clinical phenotype of sepsis.^30^ During ARDS, the pulmonary endothelial glycocalyx regulates neutrophil adhesion and lung injury^30^ and is itself damaged by inflammatory conditions such as sepsis.^31^ Therefore, the abrogation of inflammation may protect the endothelial glycocalyx structure.^32^, ^33^

Injury to the endothelial glycocalyx is thought to be an exacerbating factor of ARDS, as described above. Injury of the endothelial glycocalyx due to a disease leaves the endothelial cells vulnerable to injury and renders the patient highly susceptible to ARDS. In addition, the exposure of the cell surface receptors to the vascular lumen enables granulocytes and platelets to adhere to the endothelial cells. Consequently, the endothelial cells are injured and a thrombus forms, leading to the blockage of the blood flow (Figure 1). One of the explanative mechanisms of endothelial glycocalyx injury in several situations is the relationship between TF and TFPI. While TF, one of the most potent stimuli for rapid coagulation, is packed tightly around the arterioles and capillaries,^34^ TFPI binds to heparin sulfate, which is one of the components of endothelial glycocalyx and exists in endothelial glycocalyx.^35^, ^36^ It was previously reported that the loss of TFPI allows initiation of blood coagulation by TF in the vessel wall.^34^ It is considered that decreasing TFPI due to endothelial glycocalyx injury also promotes coagulation in the vessel.

The endothelial glycocalyx is degraded by several factors. In the clinical syndromes of systemic inflammation, including sepsis, major surgery, trauma, ischemia/reperfusion, and prolonged hyperglycemia, diffuse and persistent changes in the glycocalyx are associated with widespread endothelial dysfunction, altered permeability, and impaired oxygen and nutrient delivery to cells.^5^, ^20^, ^29^ Several previous reports have suggested associations of endothelial glycocalyx injury with surgical invasion and severe diseases such as acute kidney injury, chronic kidney disease, and cardiovascular disease.^37^, ^38^, ^39^, ^40^ In addition, chronic conditions such as diabetes,^41^ aging,^42^ and hypertriglyceridemia^43^ injure the structure of the endothelial glycocalyx and cause degradation. Consequently, patients with these conditions may be more susceptible to a rapid exacerbation of ARDS. The ability to treat and protect the endothelial glycocalyx directly would suggest an extremely important finding in this context.

## 6 WHAT CANDIDATES FOR ENDOTHELIAL GLYCOCALYX PROTECTION ARE AVAILABLE?

Previous reports indicated that an intact glycocalyx protects against endothelial disorders.^6^, ^21^ Although endothelial glycocalyx protection has been applied in specific clinical therapeutic strategies for the treatment of sepsis,^44^, ^45^ the beneficial effects of this approach remain controversial and the associated mechanisms are not yet characterized. Moreover, although antithrombin,^46^ corticosteroid,^47^ sivelestat (a neutrophil elastase inhibitor),^32^, ^33^ and antioxidant therapies^41^ appeared to protect the endothelial glycocalyx in basic experiments, these approaches remain controversial in clinical settings.^48^, ^49^ Corticosteroids reduce inflammatory damage to the endothelium in systemic sepsis.^47^ However, systemic glucocorticoid administration also increases the likelihood of secondary infection, and these drugs remain controversial in the treatment of sepsis. Antioxidant therapies may help to preserve the integrity of the glycocalyx,^41^ although definitive evidence supporting their clinical utility for the treatment of sepsis remains lacking.

Likely, the efficacies of the above‐mentioned potential therapies depend on their anti‐inflammatory effects. Inflammation is a double‐edged sword, as this reaction is part of the healing process but is harmful in excess. Accordingly, the effects of anti‐inflammatory agents against infectious diseases remain controversial, and the appropriate drug delivery methods and timing remain to be determined. This lack of an established effective and direct treatment that targets the endothelial glycocalyx emphasizes the importance of preventing endothelial glycocalyx injury. Additionally, the ability to detect an endothelial glycocalyx injury is crucial.

One study based on an experimental model of sepsis observed a considerable increase in albuminuria, a reliable marker of sepsis‐induced endothelial barrier alterations, which was presumably associated with changes to the structure of the glycocalyx.^50^ Moreover, the extent of glycocalyx injury can be estimated indirectly by the penetration of red blood cells^51^ or the serum concentration of syndecan‐1.^52^ In fact, the latter parameter was used as an endothelial injury marker in a recent clinical study.^43^, ^53^ Hyaluronic acid, a component of the endothelial glycocalyx, has also been used as a marker of glycocalyx injury.^46^ For now, early detection via a specific marker of injury remains the best means of endothelial glycocalyx protection and may enable the cure of an underlying disease.

A very recent report revealed that recombinant human thrombomodulin (rhTM) protects endothelial glycocalyx from lipopolysaccharide (LPS)‐induced pulmonary injury.^54^ This mechanism may be involved with not only the reduction of the damage associated with inflammation but also the acceleration of the biosynthesis of the glycocalyx itself. Thrombomodulin exists at the luminal surface of all vessel segments and especially at venular endothelial cell junctions,^34^ binds to thrombin to inhibit its pro‐coagulant activity, and promotes anticoagulant protein C activation. rhTM also promotes protein C activation^55^, ^56^, ^57^ and reduces the secretion of inflammatory cytokines, including interleukin‐6 and tumor necrosis factor‐α, under septic conditions.^58^ Although further experiments are required to understand how rhTM affects endothelial glycocalyx biosynthesis, it has been suggested that there is an interaction between thrombomodulin and endothelial glycocalyx thrombomodulin at least.^54^

## 7 FUTURE DIRECTIONS: COVID‐19 AND THE ENDOTHELIAL GLYCOCALYX

A patient whose pulmonary vasculature is blocked by thrombosis will not benefit from treatment with a respirator. In fact, 80% of such patients eventually die from the complications of COVID‐19. Accordingly, anticoagulation therapy and thrombosis prevention are the basis of treatment for serious COVID‐19, including ARDS, and this approach is expected to dramatically improve the symptoms of patients with serious COVID‐19. Clinicians have already begun to administer small doses of anticoagulant drugs as a precautionary measure. Many hospitals are also increasing the doses of anticoagulants administered to critically ill COVID‐19 patients. However, the risk of bleeding increases with higher doses of anticoagulant drugs. Bleeding dysfunctions observed in patients may be attributable to a hyperactive anticoagulant response.^59^ Moreover, severe COVID‐19 is also associated with fibrin degradation products and reduced platelets, which may indicate hyperfibrinolysis. A further clinical study is needed to establish an effective anticoagulant therapy strategy for COVID‐19 patients.

Likewise, endothelial glycocalyx restoration therapy may be considered as another anticoagulant strategy. While it is certainly understandable that little evidence exists with respect to COVID‐19 since these data may yet be unavailable, there are several reports on endothelial glycocalyx injury and endothelial dysfunction. In animal studies, LPS injures endothelial glycocalyx; subsequently, the endothelial cells become edematous.^26^, ^27^, ^31^ It has also been shown that experimental removal of the glycocalyx causes a dramatic rise in hydraulic permeability.^60^ In fact, the restoration of endothelial glycocalyx improves the survival in mice with sepsis.^61^, ^62^ In addition, rhTM attenuates endothelial dysfunction by endothelial glycocalyx restoration through its biosynthesis.^54^

During this COVID‐19 pandemic, many scientists are researching on SARS‐CoV‐2 and the associated host immune defenses. Undoubtedly, this work is extremely important. However, viruses often mutate, suggesting that the therapeutic strategies against COVID‐19 must also be versatile. A therapeutic strategy based on glycocalyx protection would be effective for COVID‐19 patients with both early and severe (eg, ARDS) disease. For example, a patient with comorbidity such as diabetes or hypertension would most likely exhibit an impaired glycocalyx function. Accordingly, their endothelial cells would not be fully protected and would be more susceptible to external (or internal) pathogens. In other words, the prevention of endothelial glycocalyx injury represents a useful means of systemic defense against infection. Moreover, it remains unclear whether the blood complications observed in patients with COVID‐19 are the result of a direct viral attack on the blood vessels or of an excess inflammatory immune response to the virus. Therefore, appropriate animal models that mimic not only the infection itself, but also the pattern of disease progression in humans, are needed.

In conclusion, it is important both to treat and prevent a disease. Moreover, it is important to prevent an exacerbation once a disease has been contracted. We suggest that the prevention and treatment of endothelial glycocalyx injury could potentially yield positive therapeutic effects in patients with endothelial disorders.

## AUTHORS’ CONTRIBUTIONS

HO and HT wrote the manuscript. SY, AH, and SO revised the manuscript.
